# Toward the Identification of Distinct Phenotypes: Research Protocol for the Low Back Pain Biological, Biomechanical, and Behavioral (LB^3^P) Cohort Study and the BACPAC Mechanistic Research Center at the University of Pittsburgh

**DOI:** 10.1093/pm/pnad009

**Published:** 2023-01-30

**Authors:** Nam V Vo, Sara R Piva, Charity G Patterson, Gina P McKernan, Leming Zhou, Kevin M Bell, William Anderst, Carol M Greco, Michael J Schneider, Anthony Delitto, Brad E Dicianno, Jessa Darwin, Gwendolyn A Sowa

**Affiliations:** Department of Orthopaedic Surgery, School of Medicine, University of Pittsburgh, Pittsburgh, PA 15213, United States; Ferguson Laboratory for Orthopaedic and Spine Research, Department of Orthopaedic Surgery, School of Medicine, University of Pittsburgh, Pittsburgh, PA 15261, United States; Department of Physical Therapy, School of Health and Rehabilitation Sciences, University of Pittsburgh, Pittsburgh, PA 15260, United States; Department of Physical Therapy, School of Health and Rehabilitation Sciences, University of Pittsburgh, Pittsburgh, PA 15260, United States; Department of Physical Medicine and Rehabilitation, School of Medicine, University of Pittsburgh, Pittsburgh, PA 15213, United States; Department of Health Information Management, School of Health and Rehabilitation Sciences, University of Pittsburgh, Pittsburgh, PA 15260, United States; Department of Bioengineering, Swanson School of Engineering, University of Pittsburgh, Pittsburgh, PA 15261, United States; Department of Orthopaedic Surgery, School of Medicine, University of Pittsburgh, Pittsburgh, PA 15213, United States; Department of Physical Therapy, School of Health and Rehabilitation Sciences, University of Pittsburgh, Pittsburgh, PA 15260, United States; Department of Psychiatry, School of Medicine, University of Pittsburgh, Pittsburgh, PA 15213, United States; Department of Physical Therapy, School of Health and Rehabilitation Sciences, University of Pittsburgh, Pittsburgh, PA 15260, United States; Clinical and Translational Science Institute, University of Pittsburgh, Pittsburgh, PA 15213, United States; School of Health and Rehabilitation Sciences, University of Pittsburgh, Pittsburgh, PA 15260, United States; Department of Physical Medicine and Rehabilitation, School of Medicine, University of Pittsburgh, Pittsburgh, PA 15213, United States; Department of Bioengineering, Swanson School of Engineering, University of Pittsburgh, Pittsburgh, PA 15261, United States; Human Engineering Research Laboratories, Department of Veterans Affairs, VA Pittsburgh Healthcare System, Pittsburgh, PA 15206, United States; Department of Rehabilitation and Technology, School of Health and Rehabilitation Sciences, University of Pittsburgh, Pittsburgh, PA 15260, United States; Department of Physical Medicine and Rehabilitation, School of Medicine, University of Pittsburgh, Pittsburgh, PA 15213, United States; Ferguson Laboratory for Orthopaedic and Spine Research, Department of Orthopaedic Surgery, School of Medicine, University of Pittsburgh, Pittsburgh, PA 15261, United States; Department of Physical Medicine and Rehabilitation, School of Medicine, University of Pittsburgh, Pittsburgh, PA 15213, United States

**Keywords:** phenotype, chronic low back pain, biomarkers, biomechanics, biopsychosocial

## Abstract

As a member of the Back Pain Consortium (BACPAC), the University of Pittsburgh Mechanistic Research Center's research goal is to phenotype chronic low back pain using biological, biomechanical, and behavioral domains using a prospective, observational cohort study. Data will be collected from 1,000 participants with chronic low back pain according to BACPAC-wide harmonized and study-specific protocols. Participation lasts 12 months with one required in person baseline visit, an optional second in person visit for advanced biomechanical assessment, and electronic follow ups at months 1, 2, 3, 4, 5, 6, 9, and 12 to assess low back pain status and response to prescribed treatments. Behavioral data analysis includes a battery of patient-reported outcomes, social determinants of health, quantitative sensory testing, and physical activity. Biological data analysis includes omics generated from blood, saliva, and spine tissue. Biomechanical data analysis includes a physical examination, lumbopelvic kinematics, and intervertebral kinematics. The statistical analysis includes traditional unsupervised machine learning approaches to categorize participants into groups and determine the variables that differentiate patients. Additional analysis includes the creation of a series of decision rules based on baseline measures and treatment pathways as inputs to predict clinical outcomes. The characteristics identified will contribute to future studies to assist clinicians in designing a personalized, optimal treatment approach for each patient.

## Introduction

Chronic low back pain (cLBP) has proven to be one of the most costly and difficult to treat chronic conditions of our time. cLBP is an increasingly heavy socioeconomic burden worldwide and one of the most reported musculoskeletal conditions.^1^ With the rapid growth of an aged population, and a large number of older adults who experience cLBP, improved interventions are needed to for prevention and treatment. cLBP not only results from anatomic and biochemical degeneration, but also the compounding effects of aberrant biomechanics, unhealthy lifestyle, genetics, comorbidities, and environmental and psychosocial factors. Because cLBP is multi-factorial, multi-dimensional, and highly complex, it is perhaps no surprise that traditional interventions targeted to one or a few individual contributors to back pain have been sub-optimal at best and harmful at worst. Despite the increasing costs for care, treatment outcomes and rates of disability have not improved.^2^

Funded by the National Institutes of Health (NIH) Healing Addiction Long-Term (HEAL) Initiative’s Back Pain Consortium (BACPAC) Research Program, the University of Pittsburgh Low Back Pain: Biological, Biomechanical, Behavioral Phenotypes (LB^3^P) Mechanistic Research Center (MRC) performs in-depth phenotyping of people with chronic low back pain (cLBP), using a transdisciplinary approach, to characterize individuals and provide insight into the phenotypes associated with the cLBP experience to direct targeted and improved treatments. Improved prediction capacity will facilitate the right treatment for the right patient at the right time, which could conceivably prevent the conversion to chronic opioid use that is common after failed treatment for cLBP, an important goal of the HEAL initiative.

**Figure 1.**
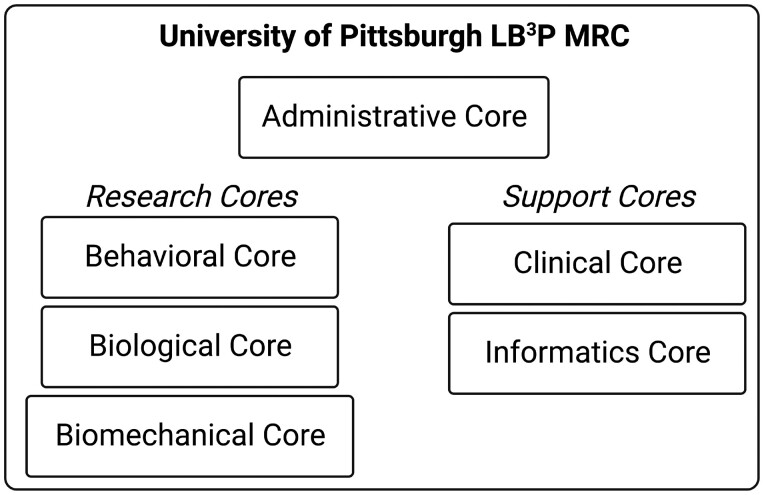
Organizational structure of the LB^3^P MRC.

Large-scale data elements collected for cLBP to date have not been well integrated into the comprehensive description of this complex condition. Traditional analytics also fail to maintain the unique relationship between distinct contributors to cLBP, particularly when pulling data elements from different sources and domains.^3–5^ Rather, the analyses need to include the interdependence of all characteristics that affect one another to address this challenging clinical enigma. The interaction of individual variables is critical in any phenotyping strategy, since in the syndrome of cLBP there is significant interaction of each contributor and the bidirectional relationships between factors predisposing individuals to chronic pain are well-established in the literature.^6^ The LB^3^P MRC’s primary goal is to define unique phenotypes in the context of response to treatment, and thereby provide the knowledge necessary to facilitate individualized treatments with greater likelihood of successful outcomes. A comprehensive pragmatic approach yields the most generalizable and clinically useful prediction information. This work is completed by the LB^3^P MRC’s three research cores (Biological, Biomechanical, and Behavioral) and three support cores (Administrative, Clinical, and Informatics) (Figure 1). The MRC's goal is to collect comprehensive datasets associated with clinical, biological, biomechanical, and behavioral characteristics of cLBP with the purpose to integrate these diverse data elements into comprehensive models. The primary objective of the study is to use the collected data to facilitate in depth phenotyping of participants to categorize types of cLBP as well as explore the impact of these phenotypes on response to different treatments. An important additional objective is to collect large datasets that will become publicly available to facilitate future hypothesis testing.

## Methods

### Study design and population

**Figure 2.**
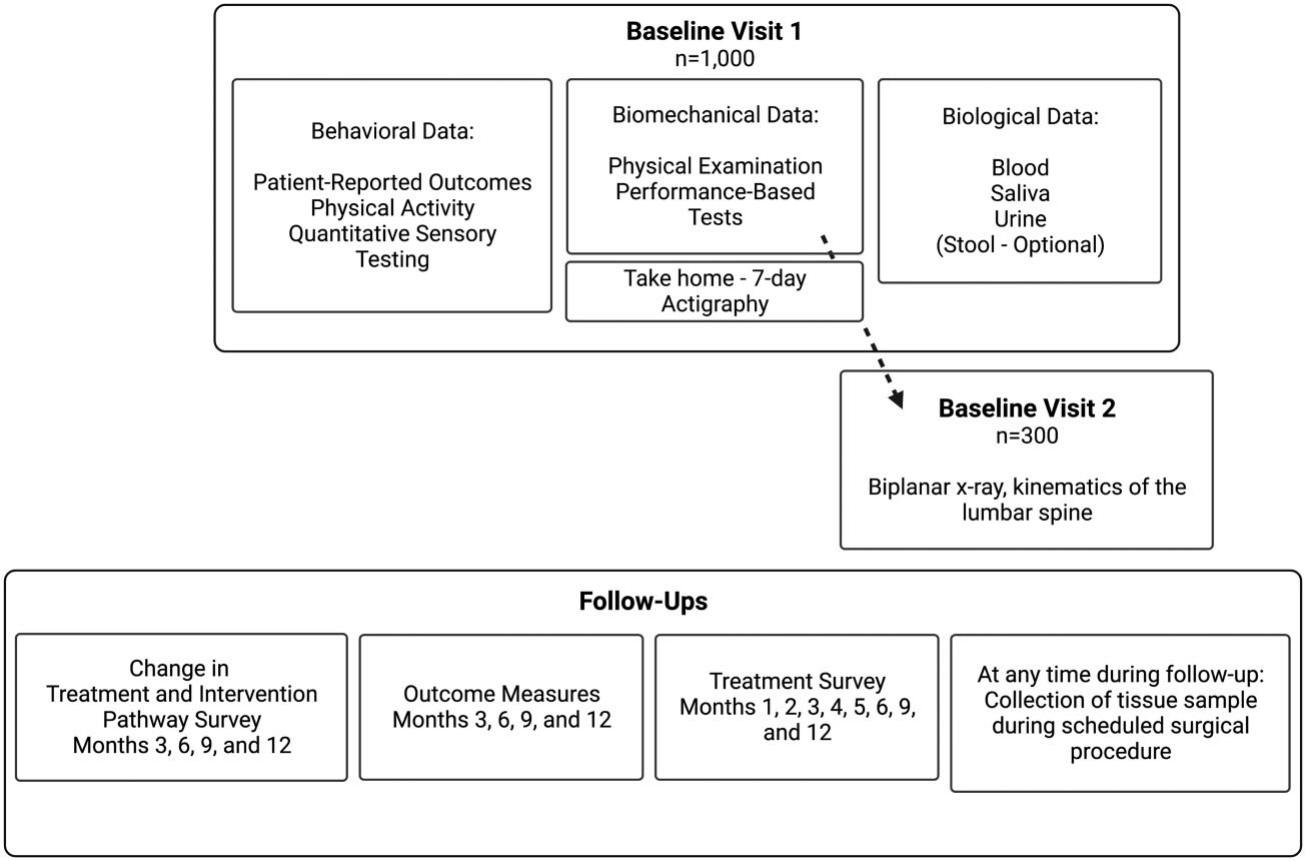
Flow of study visits and follow-ups.

The study has Institutional Review Board approval (STUDY20030093). This prospective observational cohort study is enrolling 1000 adults with cLBP for a 12-month study period (Supplemental Material, STROBE Checklist). Data are collected during treatment under the care of the participant’s clinical team and not influenced by participation in the study. The study flow is illustrated in Figure 2. Participants are recruited through routine clinical care, research registries, and public announcements.

Inclusion criteria are defined by the NIH’s definition of cLBP—pain between the inferior border of ribcage and gluteal fold for at least 3 months, resulting in pain on at least half the days in the past 6 months.^7^ Additional inclusion criteria are age ≥18years, ability to speak and understand English to complete informed consent procedures and respond to study questions, willingness to comply with all study procedures, availability for the duration of the study, and consent to the study. Participants are excluded if not identified in our affiliated electronic health record system, are currently participating in a double-blind intervention study for LBP (due to treatment intervention), or have any medical condition or characteristic that in the investigators' judgment would place them at increased risk or result in non-compliance. Participants who are participating in other treatment studies are eligible if they are aware of their treatment assignment (Supplemental Material, Treatment Pathways Form). Additional exclusion criteria apply to baseline visit 2: body mass index ≧ 35 kg/m^2^ (for image reliability), pregnancy, inability to perform flexion, extension, and side bending of their lower back, and previous multi-level lumbar fusion surgery. As the goal is to maintain an assessment of patients response to various treatments, no specific treatments or pathoanatomical diagnoses other than those that potentially place the participant at risk as outlined in the exclusion criteria will be excluded.

Screening happens over the phone and eligible participants attend an in-person baseline visit 1 consisting of biospecimen collection, patient-reported outcome (PRO) questionnaires, and a comprehensive physical exam and performance-based tests. Following the visit, participants complete a 7-day period of assessment at home with activity monitors and motion sensors.

Over 6 follow-up time points, participants completed Change in Treatment and Intervention Pathways surveys (months 1, 2, 4, and 5) and Outcome Measures and Treatment assessments (months 3, 6, 9, and 12). All follow-up data are collected electronically via emailed survey link. The study also obtains electronic health record (EHR) data from participants every 6 months.

The study also consists of 2 optional cohorts: 1) a subset of 300 participants is invited to participate in a second baseline visit involving computed tomography (CT) imaging and biplanar radiography to measure lumbar intersegmental kinematics; and 2) participants who have a clinically indicated spinal surgery during study participation are invited to donate spine tissue samples.

### Research cores

Research cores are responsible for all data collected within each of the respective domains. The variables selected for the study were based on previous data demonstrating impact on cLBP, harmonization efforts with the BACPAC consortium, and practical considerations including maximizing information while also respecting participant burden. While clearly the variables measured do not represent an exhaustive list of every potential contributor or modulator to cLBP, through measuring multiple variables across several domains within the same participant, it is anticipated that a more comprehensive picture of cLBP will be revealed.

#### Behavioral core

cLBP has a well-recognized psychological impact on the individual and their family but is poorly addressed.^8^^,^^9^ This largely relates to previous attempts to address contributors in isolation, without thoroughly understanding the multi-faceted context in which they occur.^10^^,^^11^ The relative contribution of psychosocial factors to the experience of pain and response to treatment is critical. The Behavioral Core uses assessments and measures to contextualize factors that are likely to predict variation in treatment response among patients with cLBP. Selection of assessments and measures were made based on recommendation of the BACPAC Biobehavioral Working Group (WG) and the complete rationale for biobehavioral data domains and procedures are described in detail elsewhere.

There are five data domains of interest to the Behavioral Core: 1) pain characteristics; 2) general psychosocial factors; 3) pain-related psychosocial factors; 4) lifestyle behaviors; and 5) social determinants. These data domains are collected through a battery of assessments (Table 1). The Behavioral Core also uses data from Quantitative Sensory Testing (QST), which includes 1) pressure pain threshold (algometry); 2) repeated pinprick sensation (temporal summation) on the dominant volar forearm and low back pain site; and 3) conditioned pain modulation, with pressure pain at the contralateral trapezius as the test stimulus and hand immersion in cold water (5 degrees Celsius) as the conditioning stimulus (Table 2). In response to the COVID-19 pandemic, the BACPAC baseline demographic questionnaire was amended to also include 2 questions related to COVID diagnosis and treatment.

**Table 1.** Patient-reported outcomes

Timepoints Administered	Questionnaires (Number of Questions)
BL	Demographics (MDS) (19-21)
BL, 3 mo.	History of Low Back Pain (MDS) (9)
BL, 3, 6, 9, 12 mo.	PROMIS Physical Function (6)
BL, 3, 6, 9, 12 mo.	PROMIS Anxiety symptoms (4)
BL, 3, 6, 9, 12 mo.	PROMIS Depression symptoms (4)
BL, 3, 6, 9, 12 mo.	PROMIS Fatigue (4)
BL, 3, 6, 9, 12 mo.	PROMIS Sleep Disturbance (6)
BL, 3, 6, 9, 12 mo.	PROMIS Ability to Participate in Social Roles and Activities (4)
BL, 1, 2, 3, 4, 5, 6, 12 mo.	PROMIS Pain Interference (4)
BL, 1, 2, 3, 4, 5, 6, 12 mo.	PROMIS Pain Intensity (1)
BL, 3, 6, 9, 12 mo.	PROMIS Cognitive Function (2)
BL, 3, 6, 9, 12 mo.	Patient Satisfaction with PROMIS symptom level (8)
BL, 3, 6, 9, 12 mo.	Patient Preferences regarding Outcomes (1)
BL, 3 mo.	Tobacco, Alcohol, Prescription medications, Substance (TAPS)(1-5)
BL	PROMIS Prescription Pain Medication Misuse (1–9)
BL, 1, 2, 3, 4, 5, 6, 12 mo.	Pain, Enjoyment, General Activity (PEG) (3)
BL	Michigan Body Map of pain locations (1)
BL	painDETECT (trajectories of pain, and neuropathic pain) (9)
BL, 3, 6, 9, 12 mo.	Oswestry Disability Index (ODI) (10)
BL, 6 mo.	StarT Back Tool (9)
BL	PROMIS Pain Behavior (4)
BL	Fear Avoidance Beliefs Questionnaire (FABQ) - Physical Activity (5)
BL	Chronic Pain Acceptance Questionnaire (8)
BL, 3 mo.	Pain Catastrophizing Scale (6)
BL	Interoception: Multidimensional Assessment of Interoceptive Awareness (MAIA-2) – Not Distracting Subscale (6)
BL, 3 mo.	Patient Health Questionnaire—depressive disorder screen (2)
BL, 3 mo.	Generalized Anxiety Disorder screen: GAD2 (2)
BL	Perceived Stress Scale (4)
BL	Primary Care Post-traumatic Stress Disorder Screen (history of trauma) (1–7)
BL	Financial Strain (1)
BL	Perceived Discrimination based on race/color/ethnicity, sexual orientation or gender identity (2)
BL	PROMIS General Self-Efficacy (4)
BL	PROMIS Emotional Support (4)
BL	HEAL Positive Outlook (6)
BL	Global Physical Activity Questionnaire (8–22)
3, 6, 9 mo.	Treatment Expectations: (1+)
BL, 3, 6, 9, 12 mo.	Medication Form (1+)
BL, 3, 6, 9, 12 mo.	Pregnancy (for female): (1)

BL, Baseline; mo, months; MDS, Minimum data set; PROMIS, Patient-Reported Outcomes Measurement Information System.

**Table 2.** Battery of physical tests and QST done during baseline visit 1

Category	Test
Neurologic screening	Lower Extremity Sensory Testing
	Lower Extremity Reflex Testing
	Lower Extremity Myotome Testing
	Passive Straight Leg Raise and Crossed Straight Leg Raise Tests
	Seated Slump Test for Neural Tension
Generalized joint mobility	Beighton Score
Hip joint dysfunction	Hip Scour Test
	Hip Internal Rotation Mobility Test
Lumbar spine dysfunction	Lumbar Segmental Mobility and Prone Instability Tests
	Change in Symptoms during Repeated Lumbar Movements
	Lumbar Quadrant Test
Sacroiliac joint dysfunction	Sacroiliac Distraction Test
	Sacroiliac Thigh Thrust Test
	Sacroiliac Gaenslen's Test
	Sacroiliac Compression Test
	Sacral Thrust Test
Movement control	Active Straight Leg Raise Test
	Postural Lifting Strategy Test during Floor-to-Table Load Transfer
Muscle strength and endurance	Hip Abduction Strength Test with Dynamometer
	Hip Extension Strength Test with Dynamometer
	Hip External Rotation Strength Test with Dynamometer
	Quadriceps Strength Test with Dynamometer
	Active Sit-up Test for Endurance of Abdominal Muscles
Functional performance	4-Meter Walk Test
	5 Times Sit-to-Stand Test
	Standing Balance Tests
	2-Minute Walk Test
QST, Quantitative Sensory Testing	Pain Pressure Threshold Test
	Pain Temporal Summation Test
	Conditional Pain Modulation Test
	Cold Pain Tolerance Test

Physical activity is measured using wearable devices (ActiGraph GT9X Link, Pensacola, FL). All participants are instructed in the use of the activity monitors and motion sensors to be worn at home—two accelerometry-based activity monitors (one wrist-worn, to capture sleep-related data, and one at waist level, for steps and activity counts, clipped or with a belt). Devices are worn for 7 consecutive days then returned by mail via pre-labeled envelopes. Captured data sync to cloud-based Centre Point software. Data include sleep, sedentary behavior, and light, moderate, and vigorous daily activities.

#### Biological core

**Figure 3.**
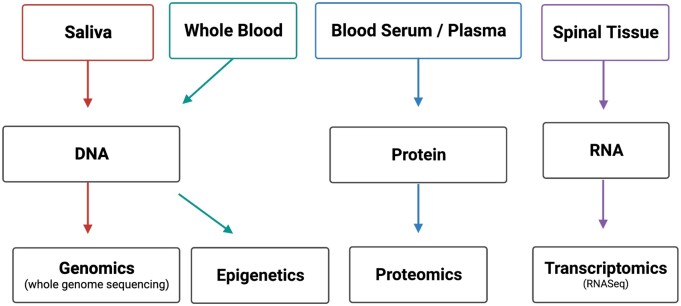
Summary schematic of biospecimen and omics analyses.

Studies show that a number of aspects of pain experience and symptomology are associated with genetics.^12–15^ Genetics therefore represent a crucial puzzle piece to elucidating the loci, progression, and potential treatment of cLBP. The Biological Core benefits from the ability to examine relevant biological mediators in the context of a vast dataset of other measures in participants with cLBP. Three classes of biomarker data are being collected: 1) saliva genomics, 2) spine tissue transcriptomics, and 3) plasma proteomics (Figure 3). Complete rationale for selecting omics analysis and description of protocols can be found elsewhere from the BACPAC Biospecimen WG and is summarized in Table 3. Also collected and stored for future analyses are urine and stool samples, the latter is done using a take home kit with a return mail package. Recently, BACPAC funded a non-cLBP control study in which 90 participants will be recruited and tested from PITT, University of California San Francisco, and Brigham Young University, totaling 270 non-cLBP subjects.

**Table 3.** Biological samples and corresponding analyses.

Biological Sample	Amount	Analysis
Blood—plasma	1 × 10 mL tube	ELISA
		MS-based proteomics
Blood—serum	1 × 10 mL tube	ELISA
		MS-based proteomics
Blood—RNA	2 × 2.5 mL tubes	RNAseq: transcriptomics
Saliva	2 × 2mL	Whole genome sequencing
Spine tissue^a^	200–1000 mg	RNASeq: transcriptomics

ELISA = enzyme-linked immunosorbent assay; MS = Mass spectrometry.

a Spine tissue samples can include ligamentum flavum, facet joint cartilage, cartilaginous endplate, and intervertebral disc nucleus pulposus and annulus fibrosus.

##### Genomic data

Saliva samples are collected using Oragene•DISCOVEROGR-500 Saliva kits. Genomic DNA is isolated from saliva. DNA libraries (average: 450 bp, range: 300-600bp) are prepared and complete DNA sequencing is performed using NovaSeq 6000 platform (Illumina) to an average target depth of 30–40× coverage (guaranteed >20×). Genome-wide association analyses are conducted using logistic regression models with additive genetic effects, between control and cLBP cohorts. In addition, DNA methylation has been shown to be indicative of changes in gene expression in patients with chronic pain, including cLBP.^16–18^ For epigenetic analysis, methylation status across over 850 000 CpG sites in the genome are assessed after bisulfite conversion of DNA isolated from whole blood aliquots prior to processing using Illumina Infinium Assay kits.

##### Transcriptomic data

Since approximately 5% of all LB^3^P patients in our system undergo a surgical procedure, we aim to collect 50 surgical samples from the full cohort. RNAs are purified from spine tissue samples. Cluster generation and 75 bp paired-read dual-indexed sequencing are performed on Illumina NextSeq 500. The libraries are normalized and pooled, then sequenced using NovaSeq6000 platform (Illumina) to an average of 50M reads. Differential expression is quantified using DESeq2. Read counts are then normalized across all samples and significant differentially expressed genes are determined. Using RNA sequencing we profile differences in gene expression among cLBP patient populations and determine how these profiles change during cLBP progression.

##### Proteomic data

Advances in proteomic methods now permit thousands of proteins to be profiled from tissue^19^ and plasma.^20^ The established label-free differential mass spectrometry (dMS) is an unbiased, robust approach to identify differences in protein expression in in vitro experiments, preclinical species,^21^^,^^22^ and humans.^23^^,^^24^ A dMS approach is advantageous because it supports large multi-level study designs that compare protein expression across multiple time points and/or treatment conditions.

The initial biomarker discovery experiments are designed to include selected groups of samples (n=∼50/group for transcriptomics and proteomics) from patients with extreme or unique clinical phenotypes. For the high pain CLBP group, all subjects had pain ≥7 and ODI ≥34, while for the low pain CLBP group all subjects had pain ≤3, ODI ≤20. This selection guaranteed a 14-point minimum separation between the two categories, thus being above the minimally clinically significant difference for CLBP, which has been indicated to be between five and 12 points.^25^^,^^26^ High-resolution Fourier transform MS is used to profile complex samples derived from serum of cLBP patients. Candidate cLBP biomarkers that are identified in these discovery proteomics experiments will be prioritized for follow-up in targeted confirmation studies using antibody/EILSA based platforms in the complete cohort.

#### Biomechanical core

It is well documented that individuals with LBP display physical impairment (eg, reduced lumbar ROM, slower movement speed, lumbopelvic rhythm) compared to asymptomatic controls.^27^ Physical impairment is determined through an objective assessment of structural limitations, and relates to anatomic loss (structure abnormality) or physiologic limitation (aberrant motion) leading to loss of ability (functional impairment).^28^^,^^29^ However, quantifying low back impairment has traditionally been qualitative—especially in the clinical setting. Biomechanical measures and rationale for selection are described elsewhere by the BACPAC Biomechanical WG.

**Figure 4.**
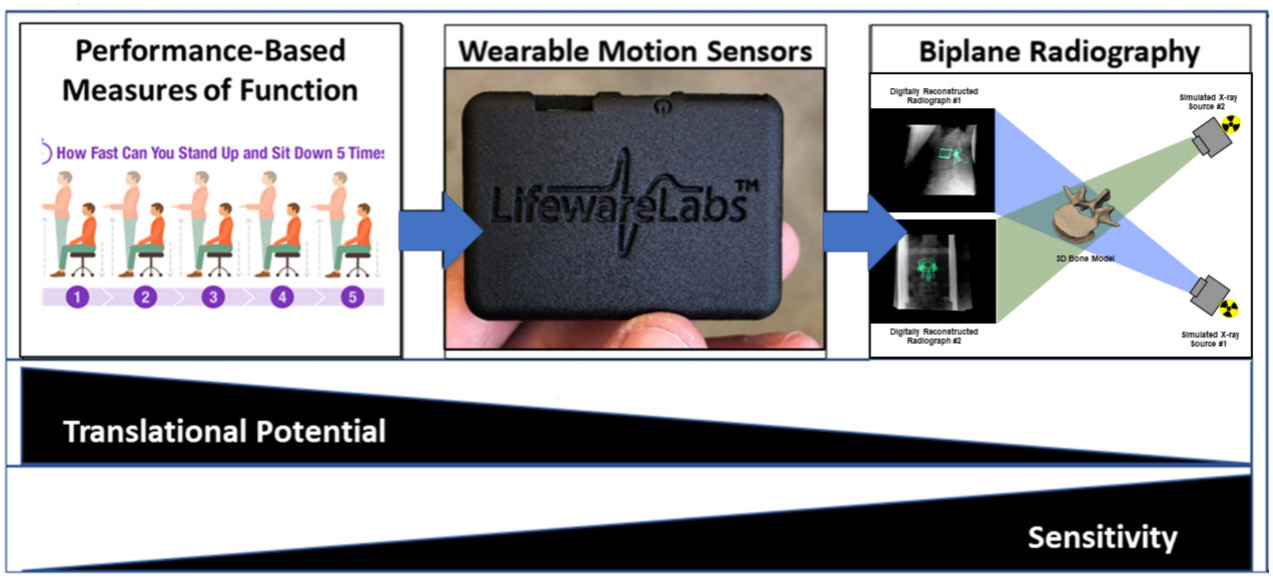
Summary of assessment completed by the Biological Core, and their translational potential and sensitivity.

The Biomechanical Core’s strength in in-depth, sophisticated laboratory-based kinematics assessments (biplane radiography) and field- and community-based assessments (wearable sensors) builds on previously published work^30–32^ linking mechanistic assessments with tracking systems that facilitate real world monitoring with greater fidelity than traditionally used approaches. Advances during this project will facilitate an improved understanding of how segmental kinematics relates to overall lumbar motion and will identify key characteristics to incorporate into the phenotyping approach. More specifically, we are utilizing ecological momentary assessment (EMA), which is a method of collecting data in the real world, in real time, and often uses mobile technology. EMA assesses the complex and dynamic nature of function and disability in a longitudinal fashion, provides data that is time- and spatially-varying in the moment it is occurring, and increases the temporal resolution of clinical assessments while limiting self-reports’ retrospective biases.^33^^,^^34^Figure 4 summarizes the methods used by the core.

##### Lumbopelvic and spine kinematics

Lumbar biomechanics are characterized during baseline visit 1 using the LB^3^P Clinical Toolbox—a custom mobile application (developed by the Health and Rehabilitation Informatics [HARI] Labs at the University of Pittsburgh) that provides digital data collection and step-by-step instructions according to BACPAC harmonized and site-specific protocols. Useability of the first version of the LB^3^P Clinical Toolbox was assessed with the mHealth App Usability Questionnaire (MAUQ, scores range between 1 and 7, with 7 being the highest positive score) resulting in a score of 5.92 (SD 0.64) indicating a highly usable system. An extensive battery of clinical exams is completed to provide an assessment of neurologic status, joint mobility, and dysfunction, neuromuscular control, strength, lumbar spine stability, and functional performance (Table 2). It should be noted that these tests also address sacroiliac joint (SIJ) dysfunction as a presumed pain generator, evidenced by the current treatments of SIJ surgical fusion to limit hypermobility and anesthetic injection for lumbopelvic pain. In addition to collecting the standard outcome metrics for each exam, pain ratings are collected for movement exams theorized to be pain provocative, and worn inertial motion sensors are used to measure lumbopelvic kinematics. Motion sensor data is collected by the LB^3^P Toolbox and is comprised of two components: 1) an in-clinic component to gather lumbopelvic kinematic data during the movement and functional performance assessments, and 2) an at-home component to capture EMA and lumbar spine kinematics over a 7-day unstructured period.

For the in-clinic component, relative orientation angles are calculated between sensors (inertial measurement units (IMUs), Lifeware Labs, LLC, Pittsburgh, PA) adhered at T1/T2, T12/L1, and the right lateral thigh relative to a sensor at L5/S1 using the anatomically aligned data. Positions, velocities, and accelerations of spinal segments and joint coordination relative to the L5/S1 sensor are calculated for the following lumbopelvic kinematic metrics: 1) maximum range of motion (ROM) of lumbar flexion/extension (F/E), axial rotation (AR) bilaterally, and lateral bending (LB) bilaterally; 2) maximum velocity of lumbar F/E, AR bilaterally, and LB bilaterally; 3) percent contribution of hip movement to F/E; and 4) lumbopelvic rhythm (segmental timing/coordination). The processed data is readily viewable in a secure clinician portal, showing participants’ spine and lumbopelvic ranges and velocities of motion and unique biomechanical markers.

The at-home component consists of two commercially available (Lifeware Labs, LLC, Pittsburgh, PA) water-resistant wearable motion sensors with onboard data logging capabilities. Data is continuously logged while the sensors are worn. Sensor data are processed to determine full-body movement patterns, gait parameters, and lumbar movement patterns. Participants also interact 3 times daily via the EMA app for a brief survey based on baseline questionnaires to capture sleep times, activity intensities, pain intensity, and interference.

##### Dynamic lumbar intervertebral motion

**Figure 5.**
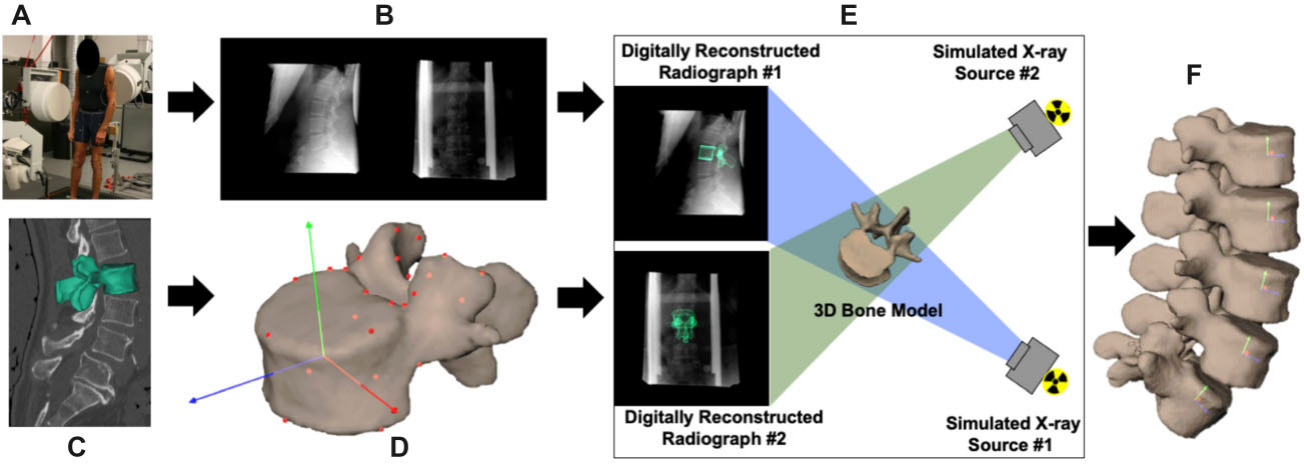
Biplane radiography data collection and processing workflow. (**A**) Participants perform flexion/extension or lumbar lifting within the biplane radiography system while (**B**) synchronized biplane radiographs are collected at 20 images per second. (**C**) CT scans are collected and used to create 3D bone models. (**D**) An anatomic coordinate system is established on each vertebra. € 3D bone kinematics are determined using a validated CT model-based tracking process11. (**F**) Intervertebral spine kinematics are calculated for each pair of synchronized biplane radiographs.

At the optional baseline visit 2, dynamic biplane radiography is used to evaluate dynamic lumbar intervertebral motion and stability during F/E, LB, and lumbar lifting. The exposure for the biplane radiography (4 milliseconds) allows for shorter exposure times compared to fluoroscopy to “freeze” the motion and eliminate motion blur. For the lumbar lifting exercise, all participants lift 20 pounds; the weight is positioned at the height of the tibial tubercles and placed 10 inches in front of the ankle joint. An axial CT scan (0.29 × 0.29 × 1.25 mm) of the lumbar spine (L1–L5) is collected. Subject-specific bone models of each vertebra are generated from the CT scans and used with stereoradiographic images to track 3D bone position and orientation for all movements performed in the system (Figure 5). Each participant performs 3 movement trials each of full ROM F/E, LB, lumbar lifting, and combined flexion plus rotation. Synchronized biplane radiographs are collected at 20 images per second for 2 seconds for each movement trial. The tracking process yields the 3D position for each lumbar vertebra during the static and dynamic movement tests. The dynamic 3D vertebral motion data is smoothed using a 4th order, low-pass Butterworth filter, with the filter frequency determined using residual analysis.^35^

Intervertebral lumbar kinematics are characterized using five parameters calculated from the continuous time-series data from each motion segment. First, the total ROM at each motion segment is determined for each movement. Second, the contribution of each motion segment to overall lumbar spine motion is determined over the continuous movements. Third, the average and peak rate of motion at each motion segment is determined for each movement. Fourth, the relationship between translation and rotation at each motion segment is evaluated. Finally, we determine the trial-to-trial repeatability of the continuous time-series kinematic data.

Hardware and software specifications, calibration and distortion correction procedures, and computational algorithms have been published previously.^36–39^

##### Utilizing an mHealth tool

**Figure 6.**
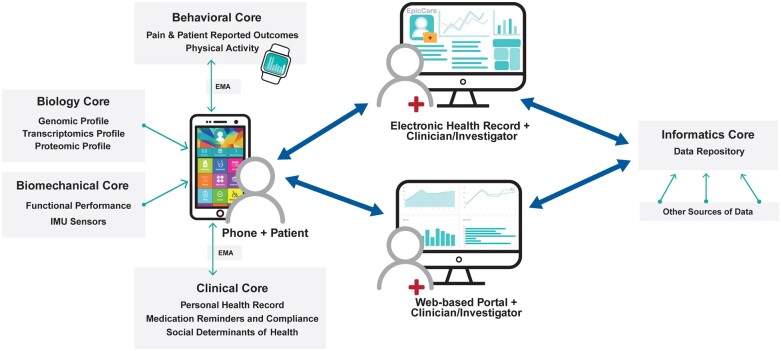
Schematic of the iMHere mobile application and its interaction with data from the various cores.

Figure 6 illustrates how a mobile health application developed by co-investigators in the Biomechanical Core will be integrated into, and leverage, the LB^3^P MRC’s work. The tool is based on a previously developed technology system^40^ and will be used by participants to collect and integrate data that contributes to the cores, as well as by clinicians and investigators involved in the research. Data flowing into the tool will also be linked to the MRC’s data repository.

### Support cores

#### Clinical core

The services provided by the Clinical Core accelerate the progress and enhance the likelihood of the MRC’s success. This core is staffed by research coordinators and physical therapists who are responsible for the in-person data collected at baseline visit 1. The core’s performance is measured by the effective enrollment and retention of the target sample and well-documented and usable datasets for analyses. Crucially, this core helps the research cores develop, standardize, and implement methods of obtaining data measures with excellent fidelity. To this end, the core developed the study Protocol, Data Safety Monitoring Plan, and Manual of Operating Procedures (MOP) to standardize all procedures and staff training for participant recruitment; administration of study forms; and data entry, management, and security. The MOP followed NIAMS guidelines and lays out the patient population description, recruitment/retention plans, protocol designs, outcome data with definitions and scoring, quality control/assurance procedures, data management and analytical techniques, sample size justification, administrative procedures, and investigators and study personnel duties/responsibilities. The MOP delineates the monitoring plans to assure participant protection and data integrity for facilitating uniformity in protocol implementation and data collection. The Clinical Core collaborated with BACPAC to refine the cLBP case definition, harmonize data integration and collection across studies, develop a questionnaire to track LBP-related treatments that is administered throughout the study, and define clinical outcome measures to be collected every 3 months (Table 1).

To achieve the study aggressive enrollment of 1000 participants, the Clinical Core uses several recruitment strategies, including: reaching out to pre-existing research cohorts of LBP; targeted outreach to neighborhoods typically under-represented in research using newsletters, postcards, and community events; research registries such as Pitt+Me^®^; and Clinical Partners. The Pitt+Me^®^ research recruitment program has over 288 000 participants and is part of the Clinical and Translational Science Institute (CTSI) at the University of Pittsburgh. It uses participants’ diagnosis codes, demographics, and/or health preferences to match participants with research studies. The Clinical Partners include 26 physicians within the University of Pittsburgh Medical Center who collectively see about 1000 patients with cLBP each month. These clinicians allow the study team to send study invitation letters on their behalf to patients with scheduled appointments identified in the EMR, which streamlines the recruitment process with minimal clinician burden.

Retention is equally crucial to the study's success. To maximize retention the Clinical Core uses strategies such as: emphasizing the importance of the coordinator-participant relationship; working closely with the clinical staff to create a sense of community; maintaining routine touch points with participants via e-mail, text messages, and phone calls; making every effort to respect the individual’s time and effort through respectful communication and flexible scheduling; and supporting participation of individuals with various types of impairments and technological challenges.

The Clinical Core is also responsible for implementing research protocols in compliance with human subjects’ protections and ensuring high quality and integrity of data. This includes developing and tracking IRB documents, ensuring all protocols and study documents are accurate and current, disseminating regulatory documents to the cores, and securing signed informed consent documents from all participants. The core monitors, adjudicates, and reports adverse events to the IRB and NIAMS/OSMB. Adverse events (AE) are collected using the National Institute of Neurological Disorders and Stroke (NINDS) Common Data Elements and graded using the Common Terminology Criteria for Adverse Events (CTCAE) based on severity (mild, moderate, severe, life-threatening, and fatal), relatedness (unrelated, unlikely, reasonable possibility, and definite) and expectedness (expected and unexpected). Adjudication of Serious AE is based on it resulting in death or being life-threatening, hospitalization, significant disability, or congenital anomaly. Criteria for Unanticipated Problem are being unexpected, related or possibly related to participation in research, and placing participant or others at greater risk. The core provides training, certification, and fidelity checks of all research protocols. Additionally, it reviews study data for regular quality control assessments and compares the data to the benchmark values, and reviews visits and forms for completeness, adverse events, data discrepancies, and protocol violations.

#### Informatics Core

**Figure 7.**
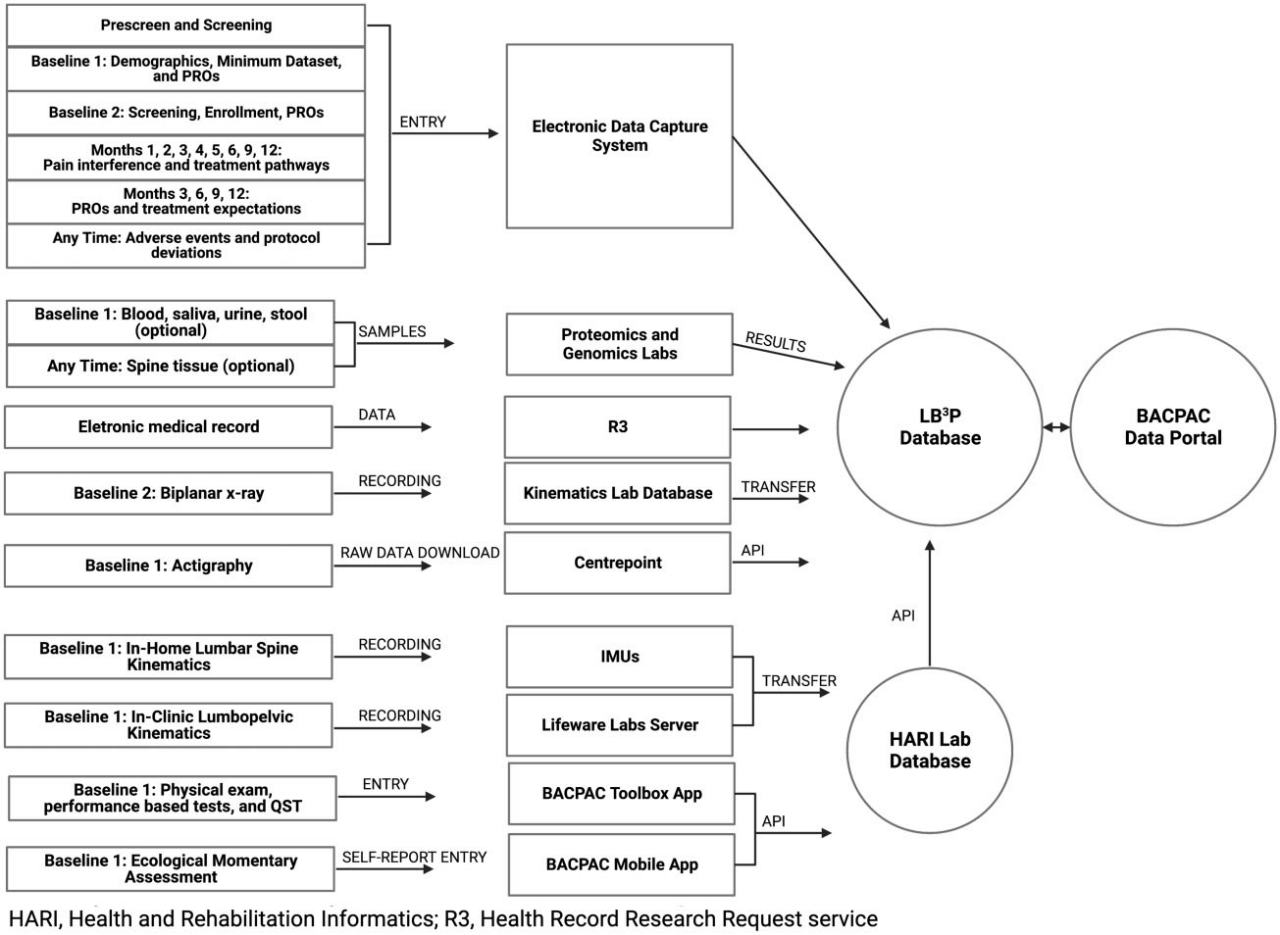
Summary of data collected, analyses, and transfer to both the site specific and consortium wide databases.

The Informatics Core’s operational aims are to ensure secure data capture that aligns with the study protocol, integrate, and harmonize data from all cores, perform analyses for the primary study objectives, and facilitate data sharing and dissemination of findings. An electronic data capture system was created to capture all screening, enrollment, demographics, participant reported measures, treatment pathway, follow-up, and adverse events. The system verifies data, has restriction on entries for discrete fields, and contains logic to minimize data entry errors and burden. The system has maximal functionality with automated communication with participants, remote follow-up via surveys, payment reports for coordinators, and email alerts for safety events. The data flow is summarized in Figure 7. An application programming interface allows integration of the BACPAC Toolbox containing all performance-based measures with the main study database. The Informatics Core is also responsible for regularly requesting and storing data exports from the UPMC Data Warehouse, the clearinghouse for clinical data.

#### Data governance and oversight

The MRC's Data Governance and Publications Committee (DGPC) consists of core leaders, the program manager, and the communications specialist. The DGPC is responsible for reporting to the corresponding governance group at the BACPAC’s central coordinating center. The committee's duties include: developing data formatting standards given the multiple sources; delineating timeframes for data processing and submission for aggregation; defining metrics for monitoring data quality; developing guidelines for data governance including stewardship, privacy, use, security, retention, and user training; and working to ensure all BACPAC data sharing obligations are fulfilled—which includes disclosing all publications that result from BACPAC funded research at LB^3^P or in collaboration with another site. The committee has a framework that facilitates approvals for manuscripts, abstracts, and presentations using the MRC data, and for ancillary study proposals or subsequent grant applications. In addition to ensuring data security, the committee opines on participant burden associated with any new proposals.

#### Administrative Core

The Administrative Core, under the PIs’ leadership, is responsible for the MRC’s operational management. Responsibilities include fiscal management, record keeping, internal and external assessment of center effectiveness, and strategic planning, as well as promoting collaboration among MRC collaborators, BACPAC, and the NIH. The core provides administrative assistance with proposal submissions, and compliance with institution specific and NIH and NIAMS requirements. Assisting the PIs are a program manager, fiscal administrator, and communications specialist. In addition to organizing internal meetings, the program manager facilitates necessary communication and meetings with NIH program directors, BACPAC investigators, institutional collaborators, and the MRC’s Advisory Board. The program manager also prepares reports at the institutional and program-wide levels.

The Administrative Core meets weekly to discuss all administrative functions for the MRC. Once a month, the leadership of all cores meet to discuss ongoing developments of the study. An annual retreat brings together all LB^3^P stakeholders where MRC leadership provide updates on recruitment/retention, and highlight accomplishments and challenges.

The Administrative Core recruited key stakeholders to serve on an external Advisory Board for the study’s duration—members were selected from patients with lived experience, the scientific community, and patient advocacy groups. The Advisory Board reviews the MRC’s progress and provides comments and recommendations to the center directors and serve to guide the planning and management of the center over the course of the award.

## Planned statistical analysis and presentation of results

Members of the Informatics Core will harmonize and translate the collected data, which will be processed and transformed from raw data (unorganized) into information (organized). An analytic dataset(s) will be developed by harmonizing file formats, naming conventions and other aspects unique to the multi-modal nature of cLBP, while using data mapping to create a common data schema. The following plan details proposed techniques to analyze high-level variable domains that will be collected—that is, PROs, performance-based measures, inflammatory markers, pain thresholds, among others. The researchers chose not to set a priori thresholds for inclusion to limit the potential for bias and/or missed findings.

### Sample size considerations

Sample size considerations for multivariate techniques such as cluster analyses are largely based on *m*, the number of variables being input into the analysis. Some recommend N = 2m or at least 500 for traditional cluster analysis. For this reason, we will conduct data reduction techniques such as principal components analysis (PCA) within domains of data prior to conducting cluster analysis. If we identify 10 phenotypes of patients, assuming equal distribution and 20% attrition at 12 months, we would have approximately 80 patients per group, which would provide good precision for outcome estimates. This would also provide approximately 80% power to detect effect sizes of at least 0.45 between any 2 groups for the continuous measures of disability, pain, or function. The effect size is defined as the difference of the means between two groups divided by the within group standard deviation. For example, a moderate to large effect is expected when comparing mean scores for disability between 2 groups. The same is true for dichotomized outcomes where the effect size is defined as h = φ1-φ2 where φ_i_=2ArcSine(√P_i_) where P is the probability of the outcome event.

### Data reduction (feature selection)

While all data domains chosen for this research are relevant to the cLBP population, it is very unlikely that all these features are equally important, especially when clustering people with cLBP into different groups and determining the relationship between these features and treatment outcomes. We will use multiple feature selection approaches, such as univariate statistics, model-based feature selection (train multiple machine learning [ML] models on the preprocessed dataset and determine the features used in those models), and iterative feature selection on these expert-identified features. PCA and spectral analysis methods for matrix-encoded features will also be applied during the procedure as data reduction and discovery of most relevant factors within a construct.

### Planned analyses

We will use traditional, unsupervised ML approaches, such as K-means clustering, to define cLBP patient phenotypes at baseline. The goal of this analysis is to categorize study participants into groups and determine the variables that differentiate these cLBP patients. This will be the foundation for characterizing the status of each given participant. Next, we will use supervised ML algorithms to examine cLBP phenotypes for critical characteristics associated with clinical response. To identify patient subgroups with shared clinical presentations, outcomes, and response to treatment, we will use neural networks (NN) and support vector machines (SVM). These methods will be applied for their ability to examine and include complex interrelationships of clinical parameters and includes these patterns as inputs in the statistical model. The structure of the combined clinical, behavioral-psychosocial, biomechanical, and biologic data are likely non-linear; therefore, we will utilize a perceptron with a multiple discriminant activation function or multiple logistic function to estimate the conditional probabilities of each class in the NN model, and the Radial Basis Function (RBF) for SVM. In addition, we will use generalized linear mixed models to assess the associations between phenotypes found at baseline, time-dependent covariates (such as different treatments), and changes in pain, disability, and function over 12 months. Finally, by exploring characteristics that are associated with the types of treatments experienced by each participant, we will identify key factors associated with variability in clinical outcomes. For this analysis we will create a series of decision rules based on the baseline measures and treatment pathways as inputs to predict the clinical outcomes, utilizing decision tree methodology, such as Chi Square Automatic Interaction Detector (CHAID). Furthermore, the patient characteristics identified in these ML algorithms can assist clinicians in designing the personalized and optimal treatment approach for patients in future studies.

### Missing data

We will monitor for missing data throughout the study. We will compare persons with missing data on domains of data (behavioral, biomechanical, and biospecimen). We anticipate minimal to no missing data at baseline, which are the main data used for the phenotyping. Should there be more than 10% missing in a domain of data, we will compare those with missing data to those without. Sensitivity analysis will be conducted with imputed values assuming missingness at random and nonignorable missingness. For the treatment pathway data and longitudinal outcomes, again we will compare those with missing follow-up data to those without to inform any conclusions we make from analyses using all available data. Again, should any set of data under investigation be missing for more than 10% of the sample, we will conduct sensitivity analyses with data imputed assuming missing at random and non-ignorable missingness.

## Conclusion

The LB^3^P MRC makes excellent use of individual research strengths at the University of Pittsburgh coalesced around a central theme and goal. By employing a pragmatic approach, the center will provide the most comprehensive assessment of patients with cLBP. The LB^3^P MRC contributes both novel datasets and robust biorepositories for use by the HEAL Initiative and the research community. It is hoped that these efforts will facilitate the design of more targeted and hence more effective and efficient, clinical trials and protocols.

## Supplementary Material

pnad009_Supplementary_Data
