# Supplementary material for: Toward the Identification of Distinct Phenotypes: Research Protocol for the Low Back Pain Biological, Biomechanical, and Behavioral (LB3P) Cohort Study and the BACPAC Mechanistic Research Center at the University of Pittsburgh
Source: Pain Med. 2023 Jan 30;24(Suppl 1):S36–47. doi: 10.1093/pm/pnad009 (PMC10403299; doi:10.1093/pm/pnad009)
Supplement: pnad009_Supplementary_Data [file pnad009_supplementary_data.zip › pnad009_Supplementary_Data/Treatment Pathways Form.docx]

**Low-Back Pain Treatment Questionnaire** *(baseline only)*

**1. In the past month, have you received surgery or a surgical procedure for your low-back pain?**

- Yes
- No

**1a. IF 1=YES**

**Did you receive a spinal fusion surgery? (Spinal fusion uses metal hardware or bone graft to permanently connect two or more vertebrae.)**

- Yes
- No

**1b. Do you have a surgery planned or scheduled for your low-back pain?**

- Yes
- No

**2. In the past month, have you received one or more injections for your low-back pain?**

- Yes
- No
- Unsure

**3. In the past month, have you taken a medication for the treatment of your low-back pain?**

- Yes
- No

**3a: IF 3=YES**

**In the past month, have you taken opioids (eg, oxycodone (Percocet), hydrocodone (Vicodin), fentanyl, tramadol (Ultram))?**

- Yes
- No

**3a.1: IF 3a=YES**

**In the past month, has your dose changed?**

- Yes, this is a new prescription/medication
- Yes, the dose increased
- Yeas, the dose decreased
- No, the dose has not changed

**3b: IF 3=YES**

**In the past month, have you taken an SSRI/SNRI (eg, paroxetine (Paxil), duloxetine (Cymbalta))?**

- Yes
- No

**3b.1: IF 3b=YES**

**In the past month, has your dose changed?**

- Yes, this is a new prescription/medication
- Yes, the dose increased
- Yeas, the dose decreased
- No, the dose has not changed

**3c: IF 3=YES**

**In the past month, have you taken Gabapentin (Neurontin) or**

**pregabalin (Lyrica)?**

- Yes
- No

**3c.1: IF 3c=YES**

**In the past month, has your dose changed?**

- Yes, this is a new prescription/medication
- Yes, the dose increased
- Yeas, the dose decreased
- No, the dose has not changed

**3d: IF 3=YES**

**In the past month, have you taken a Tricyclic Antidepressant (eg,**

**amitriptyline (Elavil), doxepin (Sinequan))?**

- Yes
- No

**3d.1: IF 3d=YES**

**In the past month, has your dose changed?**

- Yes, this is a new prescription/medication
- Yes, the dose increased
- Yeas, the dose decreased
- No, the dose has not changed

**3e: IF 3=YES**

**In the past month, have you taken a Nonsteroidal Anti-inflammatory**

**Drug (NSAID) (eg, ibuprofen (Advil, Motrin), naproxen (Naprosyn) meloxicam (Mobic), diclofenac**

**(Voltaren), celecoxib (Celebrex) or others)?**

- Yes
- No

**3e.1: IF 3e=YES**

**In the past month, has your dose changed?**

- Yes, this is a new prescription/medication
- Yes, the dose increased
- Yeas, the dose decreased
- No, the dose has not changed

**4.** **In the past month, have you received the following physical therapy, occupational therapy, or chiropractic treatments for your low-back pain?**

- Adjustment/Manipulation
- Active physical therapy or occupational therapy (e.g., supervised exercise)
- Other passive physical therapy such as ultrasound, diathermy or therapeutic massage

where you lay on a table and had a treatment administered to you

- Other
- None of the above

**4a.: IF 4=OTHER**

**What other therapy have you received in the past month?**

OPEN TEXT

**4b.: IF 4=ADJUSTMENT/MANIPULATION, ACTIVE PHYSICAL THERAPY OR OCCUPATIONAL THERAPY, OTHER PASSIVE PHYSICAL THERAPY, OR OTHER**

**How many times have you received the physical therapy, occupational therapy, or chiropractic treatments for your low-back pain** **in the past month?**

- Once
- 2 to 4 times
- 5 to 8 times
- 9 to 12 times
- 13 or more times
- Don’t know

**5. In the past month, have you participated in an exercise routine that you do on your own to**

**manage your low-back pain? *(Note, this is unsupervised exercise like an at-home aerobics***

***program or walking routine)***

- Yes
- No

**5a. IF 5=YES**

**How many days in the past week did you exercise?**

- 0 days
- 1 day
- 2 days
- 3 days
- 4 days
- 5 days
- 6 days
- 7 days

**5b. IF 5 =YES**

**Was this recommended or “prescribed” by a provider?**

- Yes
- No

**5c. If 5=YES**

**Was this prescribed or delivered via telehealth (by phone or video call)?**

- Yes
- No

**5.1. In the past month, have you participated in supervised exercise that you do with others? *(Note, this is supervised exercise like dance, pilates, group exercise with instructor or a personal trainer)***

- Yes
- No

**5.1.a. IF 5.1=YES**

**How many days in the past week did participate in supervised exercise?**

- 0 days
- 1 day
- 2 days
- 3 days
- 4 days
- 5 days
- 6 days
- 7 days

**6. In the past month, have you had acupuncture treatment for your low-back pain?**

- Yes
- No

**6a.: IF 6=YES**

**How many times have you had acupuncture for your low-back pain in the past month?**

- Once
- 2 to 4 times
- 5 to 8 times
- 9 to 12 times
- 13 or more times
- Don’t know

**7. In the past month, have you received** **mental health therapy or counseling to help you control or**

**manage your low-back pain?**

- Yes
- No

**7a. If 7=YES**

**Was this prescribed or delivered via telehealth (by phone or video call)?**

- Yes
- No

**7b.: IF 7=YES**

**How many times have you had mental health therapy or counseling in the past month?**

- Once
- 2 to 4 times
- 5 to 8 times
- 9 to 12 times
- 13 or more times
- Don’t know

**8. In the past month, have you used any mindfulness, meditation, or relaxation approaches to manage**

**your low-back pain?**

- Yes
- No

**8a. If 8=YES**

**Was this prescribed or delivered via telehealth (by phone or video call)?**

- Yes
- No

**8b.: IF 8=YES**

**How many times have you had mindfulness, meditation, or relaxation approaches in the past month?**

- Once
- 2 to 4 times
- 5 to 8 times
- 9 to 12 times
- 13 or more times
- Don’t know

**8.1. In the past month, have you used any mind/body interventions such as yoga or tai-chi to manage**

**your low-back pain?**

- Yes
- No

**8.1a.: IF 8.1=YES**

**How many times have you had** **mind/body interventions such as yoga or tai-chi in the past month?**

- Once
- 2 to 4 times
- 5 to 8 times
- 9 to 12 times
- 13 or more times
- Don’t know

**9. In the past month, have you started a diet, nutrition change, or weight loss program?**

- Yes
- No

**9a. IF 9=YES, was this recommended or “prescribed” by a provider?**

- Yes
- No

**9b. If 9=YES**

**Was this prescribed or delivered via telehealth (by phone or video call)?**

- Yes
- No

**9c.: IF 9=YES**

**Did your weight change as a result of the new diet, nutrition change, or weight loss program?**

- Lost weight
- No change
- Gained weight

**10. Since the start of the treatment (treatment), my overall pain is …**

- Very much improved
- Much improved
- Minimally improved
- No Change
- Minimally worse
- Much worse
- Very much worse

| **COVID-19 History** |  |  |
| --- | --- | --- |
| Have you been vaccinated for COVID-19? | ▢ No | ▢ Yes  If Yes, what vaccine:  ▢ Moderna or Pfizer  If Moderna or Pfizer:  ▢ I had the first dose  ▢ I had the two doses  ▢ I had a booster  ▢ Johnson & Johnson  ▢ I had the first dose  ▢ I had a booster  ▢ Don’t know |
| - Have you had a positive COVID-19 test?   *(If yes:)*  ▢ Was the test done by a health care provider?  ▢ Was the test done in a laboratory?  *(If yes:)*  ▢ Was it a PCR test?  ▢ Was it an Antigen test  ▢ Was the test done at home?   - Were you treated in a hospital overnight for COVID-19? - Did you have COVID symptoms that lasted longer than 6 weeks after the initial illness? This is sometimes called ‘long covid’, or ‘long haul’ symptoms. Symptoms may include fatigue, sleep disturbance, cough, shortness of breath or loss of smell. | ▢ No  ▢ No  ▢ No  ▢ No  ▢ No  ▢ No  ▢ No  ▢ No | ▢ Yes, in the past ▢ Yes, currently have COVID-19  ▢ Yes ▢ Unknown  ▢ Yes ▢ Unknown  ▢ Yes ▢ Unknown  ▢ Yes ▢ Unknown  ▢ Yes ▢ Unknown  ▢ Yes  ▢ Yes  If yes: Which symptoms lasted longer than 6 weeks after the initial illness? (Check all)  ▢ fatigue  ▢ sleep disturbance  ▢ cough  ▢ shortness of breath  ▢ loss of smell  ▢ pain  ▢ headache  ▢ GI distress  ▢ difficulty thinking or concentrating (sometimes referred to as “brain fog”) |
